# Circulating β-Hydroxybutyrate in Glycemic Progression and Diabetic Cardiomyopathy: Adaptive Signal or Maladaptive Substrate?

**DOI:** 10.3390/ijms27135716

**Published:** 2026-06-24

**Authors:** So Ra Kim, Byung-Wan Lee

**Affiliations:** 1 Division of Endocrinology and Metabolism, Department of Internal Medicine, Yonsei University College of Medicine, Seoul 03722, Republic of Korea; mdsrkim12@yuhs.ac; 2 Institute of Endocrine Research, Yonsei University College of Medicine, Seoul 03722, Republic of Korea

**Keywords:** β-hydroxybutyrate, ketone bodies, diabetic cardiomyopathy, type 2 diabetes, glycemic progression, mitochondrial function

## Abstract

Circulating ketone bodies (KBs), particularly β-hydroxybutyrate (β-HB), have emerged as metabolites with dual roles as both oxidative fuels and metabolic signaling molecules. Beyond serving as an alternative energy substrate, β-HB regulates diverse pathways involved in oxidative stress, inflammation, and mitochondrial function. However, the clinical implications of circulating KBs remain uncertain. This review summarizes current evidence regarding the potential role of KBs in glycemic progression and diabetic cardiomyopathy (DCM). Epidemiologic and experimental studies report conflicting associations between KB levels and the progression to hyperglycemia or type 2 diabetes, with some findings suggesting that elevated KB levels may reflect a metabolically favorable phenotype or a compensatory mechanism, whereas others indicate links to worsening glycemia. Similarly, studies in DCM have produced divergent results, with β-HB reported to improve mitochondrial function and cardiac performance in some models while contributing to metabolic inflexibility and adverse cardiac remodeling in others. We discuss potential mechanisms underlying these discrepancies and propose that the metabolic effects of β-HB are context-dependent, influenced by factors such as circulating concentration, the mode of ketosis induction, and the underlying metabolic or disease stage. Understanding these contextual determinants may help clarify whether β-HB represents an adaptive metabolic signal or a maladaptive substrate shift in cardiometabolic disease.

## 1. Introduction

### 1.1. The Metabolism of Ketone Bodies: Ketogenesis and Ketolysis

**Figure 1:**
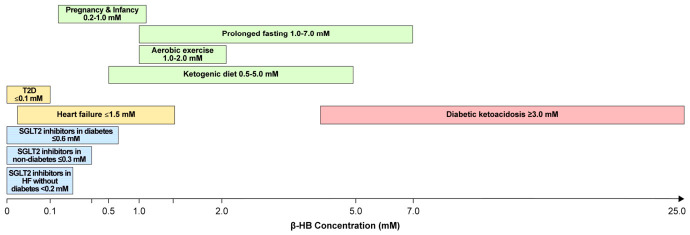
Representative circulating β-hydroxybutyrate concentrations across metabolic states. The indicated ranges are approximate and may overlap; interpretation depends on the clinical context and acid–base status. β-HB, β-hydroxybutyrate; HF, heart failure; SGLT2, sodium–glucose cotransporter 2; T2D, type 2 diabetes.

Ketogenesis occurs in hepatocyte mitochondria, whereas ketolysis involves the utilization of ketone bodies (KBs) in the mitochondria of peripheral tissues. The primary endogenous KBs, acetoacetate (AcAc) and β-hydroxybutyrate (β-HB), are synthesized from fatty acid (FA)-derived acetyl-CoA and released into the circulation. Their concentrations vary across physiological, pathophysiological, and pathological metabolic states [1] (Figure 1).

**Figure 2:**
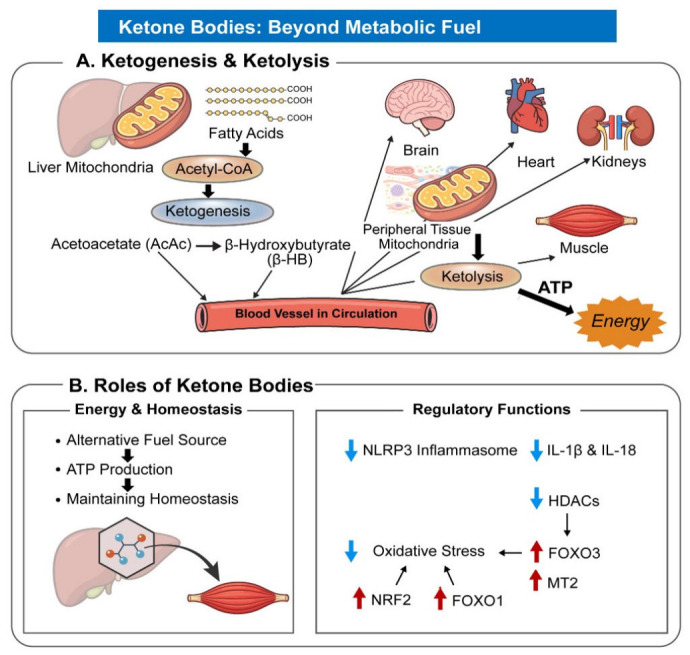
The metabolism and roles of ketone bodies. (**A**) Ketogenesis in hepatocyte mitochondria is followed by transport of ketone bodies to peripheral tissues, where they undergo mitochondrial ketolysis. (**B**) Ketone bodies serve as alternative fuel sources and generate ATP. Beyond their role as energy substrates, β-hydroxybutyrate regulates inflammation and oxidative stress via NLRP3 inflammasome inhibition, HDAC suppression, and activation of MT2, FOXO, and NRF2 signaling. β-HB, β-hydroxybutyrate; FOXO, forkhead box O; HDACs, histone deacetylases; IL-1β, interleukin-1β; IL-18, interleukin-18; MT2, metallothionein 2; NLRP3, NOD-like receptor family pyrin domain-containing 3; NRF2, nuclear factor erythroid 2-related factor 2.

Circulating KBs are transported into extrahepatic tissues via monocarboxylate transporters (MCTs). β-HB is converted back to AcAc by the enzyme 3-hydroxybutyrate dehydrogenase 1 (BDH1), which is subsequently transformed into acetyl-CoA by succinyl-CoA:3-oxoacid CoA transferase (SCOT), thereby entering the mitochondrial citric acid cycle to generate ATP. Among these key enzymes, SCOT is widely regarded as the key regulatory enzyme in KB utilization (Figure 2A).

### 1.2. Roles of Ketone Bodies

Hyperketonemia provides a glycolysis-independent source of acetyl-CoA and ATP. β-HB is considered a relatively oxygen-efficient metabolic substrate [2] that does not induce mitochondrial uncoupling—a drawback associated with increased free fatty acid (FFA) oxidation [3,4,5].

Beyond their role as energy sources, KBs, particularly β-HB, exert regulatory effects on inflammation by influencing several receptors and signaling pathways, including the NOD-like receptor family pyrin domain-containing 3 (NLRP3) inflammasome. β-HB inhibits NLRP3 inflammasome activation and the subsequent release of mature interleukin-1β (IL-1β) and interleukin-18 (IL-18) from myeloid cells [6]. β-HB also mitigates oxidative stress through multiple mechanisms. These effects, reported across various cell types, are largely attributed to β-HB-mediated protein β-hydroxybutyrylation and the inhibition of class I histone deacetylases (HDACs). In particular, β-HB enhances histone acetylation at the promoters of genes involved in oxidative stress resistance, including metallothionein 2 (MT2), and promotes Forkhead box O3 (FOXO3) expression, thereby enhancing the subsequent transcription of key antioxidant enzymes such as catalase and manganese superoxide dismutase. In addition, β-HB has been shown to activate other stress-responsive transcription factors, including FOXO1 and nuclear factor erythroid 2-related factor 2 (NRF2), which is a master regulator of cytoprotective genes involved in oxidative stress responses and detoxification pathways [7,8,9,10] (Figure 2B). These pathways are also implicated in the pathogenesis of type 2 diabetes (T2D) and diabetic cardiomyopathy (DCM), contributing to key processes such as insulin resistance, oxidative stress, and cardiac remodeling. For example, NLRP3-mediated inflammation has been linked to insulin resistance [11] and cardiac remodeling [12], whereas the FOXO and NRF2 pathways regulate metabolic and oxidative stress responses in both metabolic and cardiovascular tissues [13,14,15].

Given these diverse metabolic and signaling functions, the clinical implications of altered circulating KB levels remain incompletely investigated. In individuals without T2D, it is unclear whether elevated KBs slow the progression to hyperglycemia or the development of T2D or are simply not associated with their development. Beyond systemic glycemic outcomes, the potential role of KBs in the pathogenesis of, and therapeutic implications for, DCM also remains uncertain. This review summarizes current evidence on KBs in relation to glycemic outcomes and DCM and examines biological mechanisms that may help interpret these associations. By integrating epidemiological, experimental, and mechanistic data, this review clarifies the clinical and biological significance of KBs in cardiometabolic disease. This review focuses on β-HB in particular because it is the predominant circulating KB and, compared with AcAc, has been more extensively characterized as a bioactive signaling metabolite [16]. The pathophysiological significance of ketosis differs substantially between type 1 diabetes (T1D) and T2D. In T1D, ketosis is mainly driven by absolute insulin deficiency and may progress to pathological ketoacidosis. In contrast, in T2D, modest elevations in circulating KBs may reflect heterogeneous metabolic processes, including relative insulin deficiency, insulin resistance, altered adipose tissue lipolysis, hepatic FA oxidation, altered metabolic flexibility, and pharmacological modulation such as sodium–glucose cotransporter 2 (SGLT2) inhibition [1]. Therefore, this review focuses on the metabolic significance of circulating KBs in glycemic progression from normoglycemia to T2D and in T2D-related DCM rather than on T1D-associated ketosis or diabetic ketoacidosis.

This narrative review was based on a targeted literature search of PubMed (MEDLINE) and relevant reference lists for studies published up to May 2026. The search was conducted using combinations of terms including “β-hydroxybutyrate”, “ketone bodies”, “glycemic”, “diabetes”, and “diabetic cardiomyopathy”. Given the relatively limited and heterogeneous body of evidence, no strict restrictions were applied regarding publication period, study population, or study design. Human epidemiological and clinical studies were prioritized when available, whereas animal and mechanistic studies were included to support biological interpretation, particularly in areas where direct human evidence was limited. Studies were considered relevant if they examined circulating or urinary KB measures in relation to glycemic outcomes, T2D, or DCM. Articles were excluded if they focused primarily on T1D, diabetic ketoacidosis, non-diabetes-related ketosis without relevance to the review scope, or if they were not published in English. The selected studies were summarized narratively, with attention to study design, fasting status, KB metrics, study population, and outcome definitions. Because the available literature assessed KB status using heterogeneous measures, including blood β-HB, AcAc, total KBs, and ketonuria, this review discusses KBs more broadly where appropriate. However, conclusions are attributed specifically to β-HB when β-HB-specific data are available, whereas findings based on AcAc, total KBs, or ketonuria are interpreted as evidence for KBs in general. Given the narrative scope and heterogeneity of the available evidence, the findings were synthesized qualitatively rather than through formal risk-of-bias assessment or quantitative meta-analysis.

## 2. Association Between Circulating Ketone Bodies and Glycemic Outcomes

In T2D, insulin resistance or relative insulin deficiency impairs the ability of insulin to suppress adipose tissue lipolysis, resulting in an increased release of non-esterified fatty acids (NEFAs) into the circulation. The consequent excess delivery of FAs to the liver can enhance their oxidation and stimulate hepatic KB production, particularly when hepatic insulin sensitivity is preserved [17]. This metabolic adaptation may limit lipid accumulation and diet-induced fatty liver injury under conditions of nutrient excess [18].

**Figure 3:**
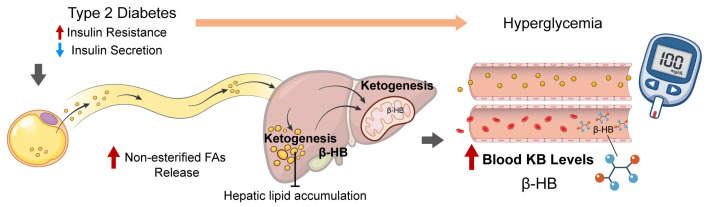
Insulin resistance or relative insulin deficiency promotes lipolysis and hepatic ketogenesis in type 2 diabetes. β-HB, β-hydroxybutyrate; FA, fatty acid; KB, ketone body.

Early in T2D, hyperinsulinemia compensates for insulin resistance and helps maintain normoglycemia. However, a progressive decline in pancreatic insulin secretion eventually leads to the failure of this compensatory mechanism, resulting in inadequate cellular responses to insulin and worsening hyperglycemia. Under conditions of insufficient insulin, the liver increases ketogenesis, predominantly β-HB, which reflects enhanced adipose tissue lipolysis and increased whole-body FA oxidation as an alternative energy source, a shift particularly evident during severe insulin deficiency [1,19,20]. Indeed, several clinical studies have reported that patients with T2D exhibit higher KB levels than individuals without diabetes, even in the absence of absolute insulin deficiency [21,22] (Figure 3).

The metabolic significance of circulating KBs may differ between established diabetes and metabolic states preceding overt diabetes. In the following sections, we examine the existing literature on the relationship between KB levels and glycemic outcomes. We first summarize longitudinal studies, which provide stronger evidence regarding temporal relationships, followed by cross-sectional studies, which should be interpreted with caution due to their inherent limitations in causal inference.

### 2.1. Favorable Associations of Circulating Ketone Bodies with Glycemic Outcomes

A 12-year prospective cohort study of 925 Korean adults (mean age of 51 years) without diabetes demonstrated that spontaneous fasting ketonuria was associated with a significantly lower risk of incident T2D compared with the absence of ketonuria (hazard ratio 0.63) [23]. Individuals with ketonuria also showed more favorable longitudinal glucose trajectories and preserved early-phase insulin secretion. These findings suggest that ketonuria may reflect a metabolically favorable phenotype characterized by lower adiposity and reduced levels of glycated hemoglobin (HbA_1C_), fasting and post-load insulin, homeostatic model assessment of insulin resistance (HOMA-IR), and triacylglycerols, along with modestly lower total energy and carbohydrate intake.

Additional longitudinal evidence from metabolite-based analyses further supports this association. In a prospective cohort of 453 Korean individuals with impaired fasting glucose (mean age, 53.8 ± 9.6 years), higher fasting circulating β-HB levels were associated with a nonsignificant trend toward a lower risk of incident T2D over a 10.9-year follow-up period [24]. Because all participants had impaired fasting glucose and Asian people often exhibit greater impairment in insulin secretion during glycemic progression [25], the authors suggested that ketogenesis in this metabolic context may represent a compensatory mechanism that mitigates the development of T2D.

Similarly, analysis of the UK Biobank cohort suggested a potential inverse association between circulating β-HB and incident T2D, whereas the association was neutral for AcAc; notably, this analysis was conducted under nonfasting conditions [26].

Finally, a large cross-sectional study of 16,523 Korean adults (mean age: 41.8 ± 8.3 years for women and 43.6 ± 7.4 years for men), conducted at a single health promotion center, reported that individuals with ketonuria had a lower prevalence of overall obesity, central obesity, and metabolic syndrome and exhibited more favorable metabolic parameters—including blood glucose, lipid levels, insulin, and blood pressure—than those without ketonuria, even after adjustment for age, smoking status, and alcohol consumption [27]. While these findings are broadly consistent with longitudinal evidence, they should be interpreted with caution due to the inherent limitations of cross-sectional designs in establishing temporal relationships.

### 2.2. Unfavorable Associations of Circulating Ketone Bodies with Glycemic Outcomes

In a large prospective cohort of normoglycemic adults [28], higher fasting KB levels were independently associated with future T2D without a significant correlation with HOMA-IR. Given the evidence that KBs exert glucose-lowering effects [29,30], the authors proposed that elevated circulating KBs may represent an early compensatory response to maintain normoglycemia in at-risk individuals.

In a Finnish cohort of men spanning the full spectrum of glucose tolerance [31], elevated circulating KB levels were also reported to predict worsening glycemia and incident T2D. In this population, the association appeared to be driven primarily by impaired insulin secretion rather than insulin resistance. Notably, higher levels of KBs were associated with greater insulin sensitivity but reduced insulin secretion, and AcAc—but not β-HB—showed an independent association with diabetes risk. Similarly, in a Finnish population-based metabolomics cohort of 11,896 young adults without diabetes at baseline [32], both AcAc and β-HB showed positive, although non-significant, associations with incident T2D over 8–15 years of follow-up.

Taken together, longitudinal evidence on the relationship between circulating KBs and progression to T2D remains limited and heterogeneous, precluding a definitive directional conclusion. Notably, differences in study design and metabolic context may partly explain these divergent findings. Variations in KB metrics (e.g., ketonuria, fasting and nonfasting circulating β-HB, AcAc, and total KBs) complicate the interpretation of available evidence. These metrics should not be considered equivalent, as β-HB and AcAc may have different metabolic and predictive implications, and ketonuria reflects urinary AcAc rather than blood β-HB. In addition, because circulating β-HB is strongly influenced by recent food intake and is generally higher under fasting conditions than in the postprandial state [33], fasting measurements may provide a more standardized assessment of basal KB metabolism. Further studies using standardized fasting blood β-HB measurements are needed to better define its association with glycemic outcomes. The inclusion of mixed populations spanning normoglycemia to overt diabetes further complicates the delineation of stage-specific effects. Favorable associations have been observed more frequently in populations with lower adiposity and relatively preserved insulin sensitivity, particularly when fasting ketonuria was used as the KB metric. In this context, fasting ketonuria may reflect preserved metabolic flexibility, whereby lower insulin levels during fasting allow a physiological shift toward FA oxidation and ketogenesis. In contrast, unfavorable associations may relate to impaired β-cell function; however, the limited and heterogeneous evidence base precludes definitive conclusions. Table 1 summarizes the studies that have investigated the association between KBs and glycemic outcomes. These findings support a context-dependent relationship and highlight the need for further mechanistic and longitudinal studies in well-phenotyped cohorts.

### 2.3. Potential Mechanisms Underlying the Favorable Glycemic Effects of Circulating Ketone Bodies

#### 2.3.1. Insulin Sensitivity

The relationship between circulating KBs and insulin sensitivity remains complex and somewhat controversial. Several studies have reported that higher circulating KB concentrations are associated with insulin resistance [34,35,36]. In contrast, other investigations have suggested that elevated KB levels may be linked to improved insulin sensitivity [31,37,38]. For example, in a cohort of 7098 young Finnish adults (mean age, 31 ± 3 years)—of whom 11% had impaired fasting glucose while the majority were normoglycemic—fasting circulating KB concentrations were inversely associated with HOMA-IR [37].

One possible explanation for these conflicting findings is that increased KB production may reflect different metabolic states depending on context. In individuals with preserved insulin sensitivity, as observed in the above-mentioned study of individuals without diabetes, which showed an inverse association between KB levels and HOMA-IR [23], modest elevations in KBs may reflect appropriate metabolic adaptation to fasting or reduced carbohydrate availability. In contrast, in insulin-resistant patients, increased KB production may arise from dysregulated adipose tissue lipolysis. As insulin resistance develops, enhanced adipose tissue lipolysis increases FA delivery to the liver, thereby stimulating hepatic ketogenesis. In this context, elevated circulating KB levels may reflect an adaptive metabolic shift aimed at maintaining energy homeostasis and mitigating metabolic stress. Supporting this concept, KBs, particularly β-HB, may exert signaling effects that improve insulin action at the cellular level. β-HB has been shown to modulate AMP-activated protein kinase (AMPK) activity and downstream metabolic regulators such as peroxisome proliferator-activated receptor γ coactivator-1α (PGC-1α). Activation of these pathways may enhance mitochondrial function and promote metabolic adaptations that could contribute to improved hepatic insulin sensitivity [39,40].

#### 2.3.2. Insulin Secretion

Regarding KBs and insulin secretion, available evidence suggests that the effects of KBs on pancreatic β-cell function are context-dependent, with acute exposure potentially enhancing insulin secretion through metabolic amplification pathways [41,42,43,44], whereas prolonged elevation of KBs may impair glucose-stimulated insulin secretion. Experimental studies have shown that β-HB alone does not directly stimulate insulin secretion but markedly potentiates insulin release when combined with glucose or monomethyl succinate in INS-1 cells and isolated rat islets. Mechanistically, β-HB enhanced mitochondrial anaplerosis by supplying acetyl-CoA for citrate synthesis and increasing short-chain acyl-CoA intermediates, thereby supporting an anaplerosis-dependent metabolic amplification pathway that augments insulin secretion downstream of Ca^2+^ signaling [44]. In contrast, other reports have described an association between elevated KB levels and reduced insulin secretion [45,46,47]. In rat pancreatic islets, a 15 h prolonged exposure to β-HB reduced glucose-stimulated insulin secretion, particularly the second phase, without altering intracellular Ca^2+^ responses. This suppression was attributed to reduced NADH supply to mitochondria via impairment of the malate–aspartate shuttle, leading to decreased ATP production [46].

Although these mechanistic findings provide important insights, their direct translation to human physiology is limited by differences between experimental models and human populations, including the definition and duration of acute versus chronic KB exposure, species-specific metabolic responses, and baseline metabolic status (e.g., glycemic control), as well as the scarcity of human mechanistic data. As discussed above, the limited longitudinal evidence suggests divergent associations with insulin secretion: ketonuria has been linked to preserved early-phase insulin secretion in individuals without diabetes [23], whereas elevated circulating KBs have been associated with reduced insulin secretion in other populations [31]. However, these findings are based on a small number of studies and should be interpreted with caution. Further mechanistic studies in humans are needed.

#### 2.3.3. Hepatic Gluconeogenesis

It is well known that ketogenesis is suppressed by obesity-associated hyperinsulinemia, leading to relative ketogenic insufficiency and hypoketonemia in obese animal models and humans [48,49,50,51,52,53]. Cotter et al. examined whether ketogenic insufficiency per se contributes to glucose dysregulation and steatohepatitis by generating a mouse model with impaired KB production using 3-hydroxy-3-methylglutaryl-CoA synthase 2 (HMGCS2) antisense oligonucleotide (ASO) treatment and characterizing its metabolic phenotype using ^13^C isotope tracing approaches [18]. HMGCS2 ASO-treated mice maintained on a low-fat diet exhibited modest but consistently elevated blood glucose levels. Mechanistically, impaired ketogenic flux redirected acetyl-CoA toward the tricarboxylic acid (TCA) cycle, thereby augmenting pyruvate-driven gluconeogenesis and facilitating cytosolic substrate availability for de novo lipogenesis. Although the degree of ketogenesis suppression in this model exceeds that typically observed clinically, the findings suggest that partial impairment of ketogenesis—particularly under conditions of increased hepatic FA influx—may similarly channel excess acetyl-CoA into gluconeogenic and lipogenic pathways.

Consistent with this finding, a study of individuals undergoing a 24 h fast quantified hepatic oxidative metabolism and gluconeogenic flux across a wide range of liver fat content [54]. In non-alcoholic fatty liver disease (NAFLD), impaired ketogenesis diverts lipid-derived acetyl-CoA toward the TCA cycle, thereby enhancing gluconeogenesis and contributing to hyperglycemia. This metabolic shift was accompanied by ~30% lower plasma NEFA and β-HB levels, potentially reflecting increased pyruvate carboxylase flux, a low glucagon-to-insulin ratio, and reduced β-hydroxybutyrate dehydrogenase activity. Similarly, in mice, ketogenic capacity declines with prolonged exposure to an obesogenic diet despite initial activation of acetyl-CoA disposal pathways during early obesity and hepatic steatosis [53].

Taken together, these findings suggest that impaired ketogenesis may disrupt hepatic acetyl-CoA disposal, thereby promoting gluconeogenesis and lipogenesis and contributing to metabolic dysregulation. This concept complements earlier observations that circulating KBs may exert context-dependent effects on insulin sensitivity and insulin secretion, highlighting the complex and stage-specific role of KB metabolism in glucose homeostasis.

## 3. Association Between Circulating Ketone Bodies and Diabetic Cardiomyopathy

### 3.1. Metabolic Dysregulation and Cardiac Remodeling in Diabetic Cardiomyopathy

T2D and obesity substantially increase susceptibility to heart failure (HF), a leading cause of death in these patients [55]. Diabetes-related structural remodeling and functional impairment of the left ventricle, which initially manifests as diastolic dysfunction, are referred to as DCM in the absence of overt coronary, valvular, hypertensive, or genetic heart disease [56,57,58]. Early metabolic abnormalities—including hyperglycemia, insulin resistance, reduced glucose utilization, and increased reliance on FA oxidation—contribute to progressive energetic insufficiency, which may ultimately lead to the development of HF with preserved or reduced ejection fraction [56,57,58,59,60]. In parallel with these metabolic alterations, hyperglycemia-driven overproduction of reactive oxygen species (ROSs) and reactive nitrogen species and activation of inflammatory pathways have emerged as central contributors to myocardial injury [61,62].

Together, metabolic inflexibility, oxidative stress, and inflammation promote cardiomyocyte apoptosis, microangiopathy, myocardial fibrosis, and impaired calcium handling [63,64], ultimately culminating in adverse remodeling and progressive cardiac dysfunction.

### 3.2. Physiological Myocardial Substrate Metabolism in the Healthy Heart

The healthy adult heart relies predominantly on mitochondrial oxidative phosphorylation to meet its high energy demand, utilizing multiple substrates including FAs, glucose-derived pyruvate, lactate, KBs, and amino acids (AAs). In the normal heart, approximately 40–60% of mitochondrial ATP is generated from FA oxidation, with the remainder derived from pyruvate, KBs, and AAs.

FAs yield the greatest ATP per carbon unit but require the highest oxygen consumption, making them the least oxygen-efficient substrates. In contrast, glucose yields more ATP per mole of oxygen consumed during oxidative phosphorylation [65]. KBs are increasingly recognized as an important myocardial fuel source. KBs are efficiently extracted by cardiac muscle, with a fractional extraction rate of approximately 40%, comparable to pyruvate and substantially higher than glucose (~2%) or FFAs (~15–20%) [3]. Myocardial KB uptake generally parallels circulating concentration [66], and when blood KB levels rise, they can become a major cardiac fuel source. In terms of oxygen efficiency, KBs are more efficient than FAs but less efficient than glucose [67,68,69].

Overall, the healthy heart exhibits marked metabolic flexibility, preferentially oxidizing FAs while retaining the capacity to utilize lactate, KBs, glucose, and branched-chain amino acids (BCAAs) depending on physiological conditions.

### 3.3. Myocardial Energy Metabolism in Non-Diabetic and Diabetic Cardiomyopathy

In a non-diabetes-specific context, the failing heart is characterized by reduced mitochondrial oxidative capacity, leading to impaired ATP production and myocardial energy deficit. This is accompanied by a loss of metabolic flexibility and substrate shifts that promote energetic inefficiency. These shifts include decreased glucose and AA oxidation and increased KB utilization. While the fasting healthy heart derives approximately 85% of ATP from FAs and 6.4% from KBs, hearts with HF with reduced ejection fraction (HFrEF) show reduced FA contribution (approximately 71%) and greater reliance on KBs (approximately 16.4%) and other substrates for ATP production. In advanced stages, myocardial ATP content may decline by up to 30% compared with the healthy heart [70,71,72,73,74,75].

In HF complicated by diabetes and obesity, myocardial substrate utilization undergoes distinct alterations. FA oxidation increases, likely driven by underlying insulin resistance, in contrast to non-diabetic HF (e.g., those with hypertensive or ischemic etiologies), in which FA oxidation declines, although this varies by disease stage. This shift reduces cardiac efficiency, indicated by lower cardiac work relative to oxygen consumption. Obese women with left ventricular hypertrophy and reduced cardiac efficiency exhibit enhanced FA utilization that correlates with the severity of insulin resistance [76]. Similarly, men with type 2 diabetic cardiomyopathy show elevated myocardial FA uptake and oxidation [77]. In experimental models of obesity and T2D, including diet-induced obese, db/db, and ob/ob mice, the heart shows predominant reliance on FA oxidation accompanied by left ventricular hypertrophy [78], diastolic dysfunction [79,80], and, in advanced stages, systolic dysfunction [81,82,83]. Conversely, myocardial glucose oxidation is reduced in HF associated with obesity and T2D. Rodent models of T2D and insulin resistance with left ventricular hypertrophy and diastolic dysfunction show marked reductions in glucose oxidation rates, a finding that is particularly common among obese and diabetic mice [84,85,86,87,88,89,90].

KB metabolism in diabetic HF appears to differ from non-diabetic HF [91,92]. In HF without consideration of diabetes status, myocardial KB oxidation is consistently increased, with approximately twofold higher utilization reported in HFrEF [4,93,94,95]. Although KBs yield more energy per carbon unit, they generate less ATP per molecule of oxygen consumed than glucose, potentially reducing energetic efficiency if KB oxidation displaces glucose oxidation. Circulating KB levels are elevated in insulin resistance and T2D; however, whether myocardial uptake and oxidation increase proportionally remains uncertain [96,97]. Although HF alone is associated with increased myocardial KB utilization, samples of cardiac tissue from patients with both HF and diabetes show repression of genes encoding key KB catabolic enzymes [91]. Transgenic models with superimposed diabetes similarly demonstrate marked downregulation of KB catabolic pathways [91,92]. In HF with diabetes, excess myocardial glucose influx drives O-GlcNAcylation of transcriptional regulators and mitochondrial proteins, suppressing electron transport chain gene expression and enzyme activity. Along with repression of KB catabolism, this limits KB utilization, promotes glycolytic intermediate accumulation, and worsens myocardial energy deficit [92].

Studies have shown that SGLT2 inhibitors reduce the risk of HF in T2D [98,99]. This effect is accompanied by modest increases in circulating β-HB, prompting the hypothesis that KB elevation contributes to improved cardiac function [100,101].

As numerous reviews have already explored the relationship between β-HB and HF of diverse etiologies [65,102,103], this section focuses specifically on DCM. Evidence regarding the role of KBs in DCM is largely derived from preclinical studies, while direct human evidence examining the effects of circulating KBs remains scarce; therefore, the existing findings should be interpreted primarily as mechanistic insights.

### 3.4. Favorable Associations of Ketone Bodies with Diabetic Cardiomyopathy

In a db/db mouse study [104], T2D mice were assigned to either a normal diet or a ketogenic diet (KD) for 8 weeks, and fasting glucose, cardiac function and morphology, mitochondrial dynamics, oxidative stress, and apoptosis were assessed. Compared with a normal diet, the KD improved glycemic control and cardiac function and attenuated cardiac remodeling. These effects were accompanied by enhanced mitochondrial function, reduced oxidative stress, decreased cardiomyocyte apoptosis, and activation of the phosphoinositide 3-kinase (PI3K)/protein kinase B (Akt) pathway in the diabetic myocardium. In isolated cardiomyocytes, β-HB exerted antiapoptotic effects by activating the PI3K/Akt pathway, thus increasing the B-cell lymphoma 2 (Bcl-2)/Bcl-2-associated X protein (Bax) ratio and decreasing caspase-3 activity.

In another db/db mouse study [105], dietary replacement of carbohydrates with a D-β-hydroxybutyrate ketone ester ((R)-1,3-butanediol monoester) for 4 weeks moderately elevated circulating β-HB levels (3.0–6.0 mM) and improved both systolic and diastolic cardiac function on echocardiography. These benefits were associated with the restoration of cardiac KB metabolic enzyme expression, enhanced mitochondrial oxygen consumption, greater resistance to oxidative/redox stress, and augmented mitochondrial biogenesis and mitophagy. The authors postulated that impaired KB oxidation is a hallmark of the diabetic heart and a potential therapeutic target. They further emphasized that the high fat content of KDs may limit their long-term suitability and that direct KB supplementation could offer a more practical alternative.

However, these findings should be interpreted with caution, as they are derived from preclinical models with heterogeneous dietary interventions (e.g., KDs versus ketone ester supplementation) and experimental conditions. Accordingly, they primarily provide mechanistic insights, and their direct clinical relevance remains to be established. In one retrospective study of patients with T2D and acute hyperglycemic crisis, ketosis without acidosis was associated with lower all-cause mortality and a lower prevalence of symptomatic HF, raising the possibility that ketogenesis helps supply the myocardium with energy under acute metabolic stress [106]; however, the effects of stress-induced ketosis differ from the chronic or modest β-HB elevations discussed in this review.

### 3.5. Unfavorable Associations of Ketone Bodies with Diabetic Cardiomyopathy

In contrast, other preclinical studies suggest that KB-related metabolic alterations may exert adverse effects on the diabetic heart under certain conditions.

In lean diabetic Goto–Kakizaki rats, long-term feeding of a low-carbohydrate, low-protein KD (62 weeks) was evaluated for its effects on cardiac metabolism and function using in vivo imaging and metabolic tracing under basal and stress conditions [107]. Although the diet improved systemic metabolic parameters, including blood glucose, triglyceride, and insulin levels, it reduced myocardial KBs and glucose oxidation while increasing FA oxidation. These metabolic alterations were associated with impaired cardiac compliance, hypertrophy, and increased oxidative stress, suggesting exacerbation of DCM, potentially driven by maladaptive cardiac metabolic modulation and lipotoxicity.

These findings highlight that prolonged or unbalanced metabolic adaptations induced by ketogenic interventions may lead to maladaptive cardiac remodeling. However, given the limited availability of human data, further human studies are needed to clarify their clinical relevance. Table 2 summarizes the studies that have investigated the association between KBs and DCM.

### 3.6. Determinants of the Context-Dependent Effects of Ketone Bodies in Diabetic Cardiomyopathy

Experimental evidence indicates that KBs exert context-dependent effects in DCM, with both protective and maladaptive consequences. In several diabetic models, elevated circulating β-HB levels improve mitochondrial function, enhance oxygen consumption, restore ketolytic enzyme expression, reduce oxidative stress, and activate prosurvival signaling pathways such as PI3K/Akt, accompanied by reduced cardiomyocyte apoptosis and improved cardiac structure and function. These findings support a role for β-HB as cytoprotective mitochondrial substrates in the diabetic myocardium. Conversely, other studies show that elevated circulating KB levels do not uniformly translate into improved myocardial energetics. KB and glucose oxidation may remain suppressed, with persistent reliance on FA oxidation, thereby reinforcing metabolic inflexibility, impaired compliance, hypertrophy, and oxidative stress. Thus, the cardiac effects of KBs in DCM appear to reflect a balance between adaptive mitochondrial signaling and unresolved defects in substrate utilization.

These discrepant findings likely arise from heterogeneity in experimental context, including KB concentration, mode of ketosis induction, dietary background, and disease phenotype.

#### 3.6.1. Concentration-Dependent Effects

First, the biological effects of β-HB appear to be concentration-dependent, differing between supraphysiological levels (e.g., >2.0–3.0 mM) and more modest elevations. In the animal studies summarized above, short-term supraphysiological increases in circulating β-HB appeared beneficial for DCM [105]; however, this conclusion should be interpreted cautiously because it is based on a very limited number of studies. Moreover, observed energetic benefits may not result directly from β-HB oxidation per se but from enhanced utilization of alternative substrates [108]. In addition, animal studies should be interpreted cautiously when β-HB concentrations exceed ranges that are practically achievable in humans, as such findings may have limited clinical relevance.

#### 3.6.2. Mode of Inducing Ketosis

Second, strategies used to elevate KBs produce distinct systemic metabolic effects. KDs, direct KB administration, and ketone ester supplementation differentially influence glucose and lipid metabolism beyond ketonemia alone. For example, maintenance of ketosis with a KD typically requires near-complete carbohydrate restriction to suppress insulin and sustain adipose lipolysis. However, high-fat diets are associated with elevated circulating lipids and can induce fatty liver and hepatic insulin resistance in mice, raising concerns regarding potential cardiovascular risk in patients with T2D [109]. Moreover, variability in macronutrient composition—particularly fat and protein content—can substantially influence cardiac and systemic metabolism, complicating comparisons across studies and limiting clinical translation. In contrast, supplementation with a pro-ketone compound such as D-β-hydroxybutyrate-(R)-1,3-butanediol monoester (ketone ester) increases plasma β-HB without requiring a high-fat diet and does not elevate circulating FFAs [110,111]. Thus, divergent cardiac outcomes cannot be attributed solely to KB levels and should be interpreted in the context of how ketonemia is induced.

#### 3.6.3. Disease Stage

Finally, disease stage critically modifies myocardial responses to KBs. Across the previously discussed studies, baseline cardiac function reflected different stages of DCM. Db/db mice with reduced systolic function at baseline showed improved cardiac function following KB-based interventions (KD or ketone ester) [104,105], whereas diabetic rats with relatively preserved systolic function (although pre-intervention cardiac function was not reported, chow-fed diabetic controls showed preserved resting EF after long-term feeding) worsened when treated with a KD [107]. However, evidence is currently insufficient to conclude that KBs are beneficial in advanced DCM but harmful in early-stage disease. Since myocardial substrate handling, mitochondrial reserve, and structural remodeling may vary across the spectrum from uncomplicated diabetes to advanced HF, whether KB exposure elicits adaptive or maladaptive responses requires further investigation. Furthermore, the existing evidence is largely limited to animal studies, with a notable lack of human data.

### 3.7. Effects of Sodium–Glucose Cotransporter 2 Inhibitor-Induced Ketonemia on Diabetic Cardiomyopathy: Signaling Modulators Rather than Bioenergetic Fuel

SGLT2 inhibitors induce glycosuria and lower insulin, thereby stimulating lipolysis and FFA release. Although hepatic ketogenesis physiologically occurs during fasting [112], SGLT2 inhibitors promote ketogenesis even in a fed state [19].

In a randomized, double-blind, placebo-controlled crossover trial, individuals with T2D treated with empagliflozin for 4 weeks underwent PET/CT assessment of myocardial substrate utilization, oxygen consumption, and perfusion [113]. Empagliflozin increased circulating β-HB without altering absolute myocardial FA uptake or oxidation, while myocardial glucose uptake significantly declined by 57%. Increased KB delivery appeared to substitute for glucose rather than less oxygen-efficient FAs. Given the minimal contribution of glucose to cardiac energetics in the postabsorptive state in T2D [114], this substrate shift was quantitatively small and did not improve myocardial oxygen consumption or external efficiency, refuting a major energetic role of KBs in SGLT2 inhibitor-mediated cardioprotection [3]. Meanwhile, in rodent models of diabetes, the hearts of untreated db/db mice exhibited markedly reduced glucose and KB oxidation, increased FA oxidation, and diminished cardiac ATP production. Despite hyperketonemia, treatment with the SGLT2 inhibitor empagliflozin did not enhance myocardial KB utilization, and although increasing KB availability raised ATP production, it did not improve cardiac efficiency, indicating that KB oxidation was not responsible for functional recovery. Instead, empagliflozin improved cardiac energy status, primarily by restoring glucose oxidation and increasing overall ATP generation [108].

Clinically, SGLT2 inhibitors raise β-HB to ~0.6 mM in patients with diabetes [115], yet they raise β-HB to just ~0.3 mM in patients without diabetes and <0.2 mM in those with HF without diabetes [116] (Figure 1). Nevertheless, the cardiovascular benefits of SGLT2 inhibitors are consistent irrespective of diabetic status. Importantly, as discussed in Section 3.4, even in experimental models with relatively high circulating β-HB levels, many of the reported cardioprotective effects have been linked to signaling and stress-adaptive pathways rather than direct energetic effects alone. Therefore, even modest ketonemia induced by SGLT2 inhibitors may exert cardioprotective effects through β-HB-mediated signaling and stress-adaptive pathways despite substantially lower circulating β-HB concentrations. Given the modest degree of ketonemia induced by SGLT2 inhibitor treatment and the reduced expression of ketolytic enzymes in the diabetic myocardium, the contribution of elevated circulating β-HB to the cardioprotective effects of SGLT2 inhibitors appears limited. This limited role is likely mediated by the SGLT2 inhibitors acting as nutrient-deprivation signaling modulators rather than bioenergetic fuel. In vitro and ex vivo evidence demonstrates that β-HB provides direct cardioprotection independent of ATP-yielding oxidation [102]. Cytosolic β-HB modulates nutrient- and energy-sensing pathways by activating Sirtuin 3 (SIRT3) and AMPK while inhibiting mechanistic target of rapamycin (mTOR) in the failing heart. Thus, ketonemia enhances autophagic flux and stress adaptation in the failing heart, primarily through metabolic signaling rather than improved bioenergetics.

Overall, therapeutic targeting of KB metabolism in DCM should be interpreted beyond the simple elevation of circulating β-HB levels. SGLT2 inhibitors, KDs, and ketone ester supplementation may exert distinct effects depending on the mode of ketosis induction and the relative contribution of β-HB as a fuel versus a signaling metabolite. Further human studies are required to determine whether these approaches can improve myocardial metabolism, attenuate oxidative stress and inflammation, or alter the clinical course of DCM.

## 4. Conclusions

**Figure 4:**
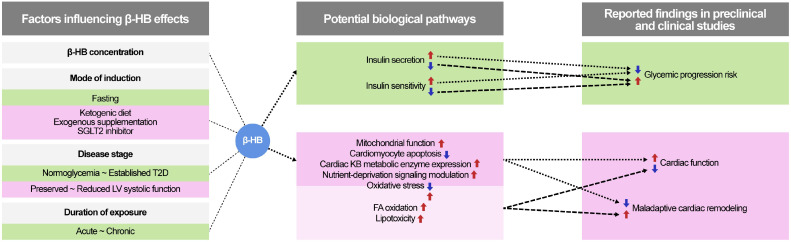
Proposed schematic summarizing factors that may influence the context-dependent effects of circulating β-hydroxybutyrate on glycemic outcomes and diabetic cardiomyopathy. This schematic is based on currently available evidence and is not intended to depict exhaustive or definitive mechanistic relationships. On the right side, dotted arrows indicate favorable/adaptive associations, whereas dashed arrows indicate unfavorable/maladaptive associations. β-HB, β-hydroxybutyrate; FA, fatty acid; KB, ketone body; LV, left ventricle; SGLT2, sodium–glucose cotransporter 2; T2D, type 2 diabetes.

Current evidence linking circulating KBs, particularly β-HB, to dysglycemia and DCM remains limited and heterogeneous, with studies suggesting both potentially adaptive and maladaptive associations. Beyond serving as an oxidative substrate, β-HB functions as a signaling metabolite whose effects depend on concentration, mode of induction, and underlying disease phenotype (Figure 4). Future studies should more clearly define the conditions under which β-HB exerts adaptive versus maladaptive effects in both glycemic progression and DCM. Key priorities include determining the optimal circulating β-HB concentration range and adopting standardized fasting blood β-HB measurements to better assess basal KB metabolism and improve comparability across studies. Future studies should also longitudinally track β-HB profiles and KB flux across disease stages to clarify stage-specific effects from early dysglycemia to established diabetes and from early cardiac metabolic dysfunction to overt DCM. In glycemic progression, well-phenotyped longitudinal cohorts are needed to clarify whether elevated β-HB reflects adaptive metabolic flexibility, compensatory ketogenesis, or early metabolic deterioration associated with altered insulin sensitivity and β-cell function. In DCM, mechanistic clinical studies incorporating myocardial substrate utilization, ketolytic enzyme expression or activity, mitochondrial function, and inflammatory or oxidative stress markers will be essential, particularly to distinguish SGLT2 inhibitor-induced ketonemia from the direct effects of KDs or KB supplementation.

## Figures and Tables

**Table 1:** Summary of human studies evaluating ketone bodies and glycemic outcomes.

Reference	Population	Study Design	KB Metrics	KB Levels	Findings Associated with Elevated KBs	Proposed Mechanism
Kim et al. [23]	925 adults without diabetes	Prospective cohort	Fasting ketonuria	Presence vs. absence	Lower risk of incident T2D (HR 0.63; 95% CI 0.41, 0.97)	Preserved early-phase insulin secretion, lower adiposity, reduced HOMA-IR, and lower total energy and carbohydrate intake
Bae et al. [24]	453 individuals with impaired fasting glucose	Prospective cohort	Fasting serum β-HB	≥0.05 vs. <0.05 mM	Trend toward lower risk of incident T2D (HR 0.70; 95% CI 0.47, 1.04)	Compensatory ketogenesis in impaired insulin secretion
Bragg et al. [26]	65,684 individuals, UK Biobank participants without diabetes	Prospective cohort	Nonfasting plasma β-HB (NMR-based)	Mean 0.01 ± 0.01 mM ^#^	Trend toward lower risk of incident T2D (HR 0.98; 95% CI 0.92, 1.05)	Not reported
Joo et al. [27]	16,523 adults undergoing health screening	Cross-sectional	Fasting ketonuria	Presence vs. absence	Lower adiposity, lower blood glucose and insulin levels	Possible preserved fat oxidation capacity
Szili-Torok et al. [28]	3307 individuals with neither diabetes nor impaired fasting glucose	Prospective cohort	Fasting plasma KBs (AcAc, β-HB, acetone) (NMR-based)	Fasting KB tertiles: <0.153, 0.153–0.212, and >0.212 mM	Incident T2D rates increased across KB tertiles (1.8%, 4.3%, and 5.4%, respectively)	Early compensatory response to maintain normoglycemia
Mahendran et al. [31]	9398 Finnish men without diabetes (METSIM cohort); 4335 underwent 5-year follow-up	Prospective cohort	Fasting serum AcAc and β-HB (NMR-based)	Mean AcAc 0.06 ± 0.04 mM and mean β-HB 0.14 ± 0.11 mM; highest quartile vs. lower 3 quartiles ^#^	Elevated AcAc, but not β-HB, was associated with incident T2D (adjusted OR 1.32; 95% CI 1.00, 1.74 for AcAc vs. 1.03; 95% CI 0.77, 1.36 for β-HB)	Impaired insulin secretion
Ahola-Olli et al. [32]	11,896 young adults without diabetes from four Finnish cohorts	Prospective cohort	Fasting/partially fasting serum AcAc and β-HB (NMR-based)	Mean AcAc 0.062 ± 0.049 mM and mean β-HB 0.18 ± 0.14 mM ^#^; ORs per 1-SD increase in log-transformed metabolite concentration	AcAc and β-HB showed trends toward higher risk of incident T2D (OR 1.09; 95% CI 0.98, 1.20 for AcAc vs. 1.10; 95% CI 0.98, 1.22 for β-HB)	Not reported

^#^ Only the mean levels of the overall cohort were reported. AcAc, acetoacetate; β-HB, β-hydroxybutyrate; CI, confidence interval; HOMA-IR, homeostatic model assessment of insulin resistance; HR, hazard ratio; KB, ketone body; NMR, nuclear magnetic resonance; OR, odds ratio; SD, standard deviation; T2D, type 2 diabetes.

**Table 2:** Summary of animal studies evaluating ketone bodies and diabetic cardiomyopathy.

Reference	Animal Model	Baseline Heart Function	KB Induction Strategy	β-HB Levels vs. Control	Findings Associated with Elevated KBs	Proposed Mechanism	Interpretation
Guo et al. [104]	8-week-old male db/db mice (C57BL/KsJ)	Impaired cardiac function (decreased LVEF and increased left ventricular internal dimension)	8 weeks of ketogenic diet	Myocardial β-HB: 1.08 ± 0.11 vs. 0.46 ± 0.04 mM	Improved cardiac function (LVEF), reduced cardiac fibrosis and heart weight	1. Activation of the PI3K/Akt pathway, which inhibits cardiomyocyte apoptosis 2. Mitochondrial dynamics: increased L-Opa1/S-Opa1 ratio, reduced fission-related signaling, and improved mitochondrial respiratory control ratio and ATP content 3. Antioxidant effect: reduces ROS and MDA levels while increasing MnSOD activity	Adaptive
Thai et al. [105]	10-week-old male db/db mice (C57BLKS/J)	Diastolic and systolic dysfunction	4 weeks of ketone ester diet (D-β-hydroxybutyrate-(R)-1,3 butanediol monoester, equicalorically replacing carbohydrates)	Plasma β-HB: 4.36 ± 0.32 vs. 0.45 ± 0.08 mM	Improved systolic and diastolic function, improved mitochondrial respiration	Restoration of cardiac KB metabolic enzyme expression, enhanced mitochondrial oxygen consumption, augmented mitochondrial biogenesis and mitophagy, greater resistance to oxidative/redox stress	Adaptive
Abdurrachim et al. [107]	12-week-old male lean diabetic Goto-Kakizaki rats	Pre-intervention cardiac function not reported; chow-fed diabetic controls showed preserved resting EF after long-term feeding	62 weeks of low-carbohydrate low-protein ketogenic diet	Fasting blood β-HB: 1.6 ± 0.2 vs. 0.6 ± 0.3 mM	Lowered myocardial KB oxidation, increased cardiac hypertrophy, increased preload, reduced cardiac compliance, lower glucose oxidation, enhanced stress-induced glycolysis, and trends toward increased myocardial lipid and collagen content	1. Reduced myocardial KB oxidation: lower glutamate production from hyperpolarized AcAc, lower SCOT activity, and lower cardiac BDH1 expression 2. Metabolic substrate imbalance: reduced glucose oxidation and enhanced fatty-acid-related remodeling 3. Lipotoxicity/oxidative stress: trends toward increased cardiac triglyceride and collagen content, with increased catalase expression as a marker of oxidative stress	Maladaptive

AcAc, acetoacetate; Akt, protein kinase B; BDH1, 3-hydroxybutyrate dehydrogenase 1; β-HB, β-hydroxybutyrate; EF, ejection fraction; KB, ketone body; L-Opa1, long-form optic atrophy 1; LVEF, left ventricular ejection fraction; MDA, malondialdehyde; MnSOD, manganese superoxide dismutase; PI3K, phosphoinositide 3-Kinase; ROS, reactive oxygen species; SCOT, succinyl-CoA:3-oxoacid CoA transferase; S-Opa1, short-form optic atrophy 1.

## Data Availability

No new data were created or analyzed in this study. Data sharing is not applicable to this article.

## Abbreviations

The following abbreviations are used in this manuscript:

AAs	Amino acids
AcAc	Acetoacetate
Akt	Protein kinase B
AMPK	AMP-activated protein kinase
ASO	Antisense oligonucleotide
Bax	Bcl-2-associated X protein
BCAA	Branched-chain amino acid
Bcl-2	B-cell lymphoma 2
BDH1	3-hydroxybutyrate dehydrogenase 1
β-HB	β-hydroxybutyrate
CI	Confidence interval
DCM	Diabetic cardiomyopathy
EF	Ejection fraction
FA	Fatty acid
FFA	Free fatty acid
FOXO	Forkhead box O
HbA_1C_	Glycated Hemoglobin
HDACs	Histone deacetylases
HF	Heart failure
HFrEF	Heart failure with reduced ejection fraction
HMGCS2	3-hydroxy-3-methylglutaryl-CoA synthase 2
HOMA-IR	Homeostatic model assessment of insulin resistance
HR	Hazard ratio
IL-1β	Interleukin-1β
IL-18	Interleukin-18
KB	Ketone body
KD	Ketogenic diet
L-Opa1	Long-form optic atrophy 1
LVEF	Left ventricular ejection fraction
MCTs	Monocarboxylate transporters
MDA	Malondialdehyde
MnSOD	Manganese superoxide dismutase
mTOR	Mechanistic target of rapamycin
MT2	Metallothionein 2
NAFLD	Non-alcoholic fatty liver disease
NEFA	Non-esterified fatty acids
NLRP3	NOD-like receptor family pyrin domain-containing 3
NMR	Nuclear magnetic resonance
NRF2	Nuclear factor erythroid 2-related factor 2
OR	Odds ratio
PGC-1α	Peroxisome proliferator-activated receptor γ coactivator-1α
PI3K	Phosphoinositide 3-Kinase
ROS	Reactive oxygen species
SCOT	Succinyl-CoA:3-oxoacid CoA transferase
SD	Standard deviation
SGLT2	Sodium–glucose cotransporter 2
SIRT3	Sirtuin 3
S-Opa1	Short-form optic atrophy 1
TCA	Tricarboxylic acid
T1D	Type 1 diabetes
T2D	Type 2 diabetes
